# Ancient DNA evidence for the ecological globalization of cod fishing in medieval and post-medieval Europe

**DOI:** 10.1098/rspb.2022.1107

**Published:** 2022-10-26

**Authors:** Lourdes Martínez-García, Giada Ferrari, Angélica Cuevas, Lane M. Atmore, Begoña López-Arias, Mark Culling, Laura Llorente-Rodríguez, Arturo Morales-Muñiz, Eufrasia Roselló-Izquierdo, Juan Antonio Quirós, Ricard Marlasca-Martín, Bernd Hänfling, William F. Hutchinson, Kjetill S. Jakobsen, Sissel Jentoft, David Orton, Bastiaan Star, James H. Barrett

**Affiliations:** ^1^ Centre for Ecological and Evolutionary Synthesis (CEES), Department of Biosciences, University of Oslo, Oslo 0315, Norway; ^2^ Royal Botanic Garden Edinburgh, Edinburgh EH3 5NZ, UK; ^3^ Laboratorio de Arqueozoología LAZ-UAM, Universidad Autónoma de Madrid, Madrid 28049, Spain; ^4^ Evolutionary Biology Group, Department of Biological Sciences, University of Hull, Hull HU6 7RX, UK; ^5^ Laboratory for Archaezoological Studies, Faculty of Archaeology, University of Leiden, Leiden 2311 EZ, The Netherlands; ^6^ Department of Geography, Prehistory and Archaeology, University of the Basque Country, Vitoria-Gasteiz 48940, Spain; ^7^ Posidonia s.l. Avd. Sant Jordi, Eivissa 07638, Spain; ^8^ Institute for Biodiversity and Freshwater Conservation, UHI-Inverness, Inverness, UK; ^9^ BioArCh, Department of Archaeology, University of York, York, UK; ^10^ Department of Archaeology and Cultural History, NTNU University Museum, Norwegian University of Science and Technology, Trondheim 7012, Norway

**Keywords:** cod trade, historical ecology, marine fisheries, zooarchaeology, biological source, genomics

## Abstract

Understanding the historical emergence and growth of long-range fisheries can provide fundamental insights into the timing of ecological impacts and the development of coastal communities during the last millennium. Whole-genome sequencing approaches can improve such understanding by determining the origin of archaeological fish specimens that may have been obtained from historic trade or distant water. Here, we used genome-wide data to individually infer the biological source of 37 ancient Atlantic cod specimens (*ca* 1050–1950 CE) from England and Spain. Our findings provide novel genetic evidence that eleventh- to twelfth-century specimens from London were predominantly obtained from nearby populations, while thirteenth- to fourteenth-century specimens were derived from distant sources. Our results further suggest that Icelandic cod was indeed exported to London earlier than previously reported. Our observations confirm the chronology and geography of the trans-Atlantic cod trade from Newfoundland to Spain starting by the early sixteenth century. Our findings demonstrate the utility of whole-genome sequencing and ancient DNA approaches to describe the globalization of marine fisheries and increase our understanding regarding the extent of the North Atlantic fish trade and long-range fisheries in medieval and early modern times.

## 1. Introduction

The expansion of long-range fish trade, not least of Atlantic cod (*Gadus morhua*), has partly driven the development of urbanized market economies across European societies during the last millennium [1–3]. The importance of this trade is well documented by historical sources from the fourteenth century and can be glimpsed in anecdotal historical records and archaeological evidence from the late eleventh, twelfth and thirteenth centuries [4,5].

Ancient DNA (aDNA) and stable isotopes have previously shown the early transport of air-dried Arctic Norwegian cod (*stockfish*) to Haithabu in Germany by *ca* 1066 CE [6,7]. This exchange developed into a major and wide-ranging Atlantic cod trade across medieval northern Europe, linking towns in Scandinavia, Germany, England and the Low Countries (e.g. Bergen, Lübeck, King's Lynn, London and Deventer) [6–8]. In the Iberian Peninsula, the northern ports were developed as strategic trading posts for receiving and distributing luxury and foreign products from both the Mediterranean and northern Europe [9,10]. As a consequence, distant-water fisheries and fish trade along the Atlantic coast, from Sevilla to western Ireland and Flanders, started to receive more interest within the Iberian market [11,12]. Subsequently, post-medieval European expansion to the western Atlantic, especially to Newfoundland, linked the above-mentioned northern and Iberian networks into competing and sometimes complementary long-range fisheries that were sources of both food and wealth [1,13]. For example, seventeenth-century English catches from Newfoundland were often traded to southern Europe, in an economically significant triangular trade that also entailed salt and wine [14].

Tracing the origin of Atlantic cod specimens harvested for these medieval and post-medieval trade networks contributes to our understanding of economic history and historical ecology. Historical and archaeological sources have revealed the extension of distant-water fisheries and trading networks through time and space [8,13]. However, the geographical and biological resolution of text-based and archaeological sources is often limited, and the level of detail in such sources often decreases with time depth [15]. Determining the biological origin of archaeological bone assemblages of species such as Atlantic cod can therefore provide important information about the populations targeted through distant-water fishing and/or trade. Since archaeological cod bones can represent local or long-distance (even intercontinental) fishing, it is important to distinguish between source populations. Thus, there has been an increased interest in the use of aDNA and stable isotope methods to identify the origin of archaeological remains to trace the development of the globalization of marine fisheries [6,16–20]. Here, we use novel whole-genome aDNA approaches to greatly improve the spatial specificity and resolution regarding the inference of source populations of archaeological Atlantic cod bones [21].

We assess the biological origin of 37 Atlantic cod specimens from medieval England (London) and post-medieval Spain (Barcelona, Álava (Castillo de Labastida), Madrid and Sevilla) using low-coverage genome-wide data. We genetically assign such specimens according to patterns of spatial genome-wide differentiation among modern populations of Atlantic cod [22–25]. We specifically investigated significant differentiation in polymorphic chromosomal inversions (i.e. LG1, LG2, LG7 and LG12) [6,26,27] that are associated with migratory behaviour and temperature clines [22,27–30]. Their genetic differentiation can therefore indicate the assignment of specimens towards a particular geographical area [6,31]. Through these methods, we aim to distinguish source populations with improved discriminating power in relation to previous stable isotope and aDNA approaches [6,15,16].

## 2. Materials and methods

### (a) Sample collection

**Figure 1.**
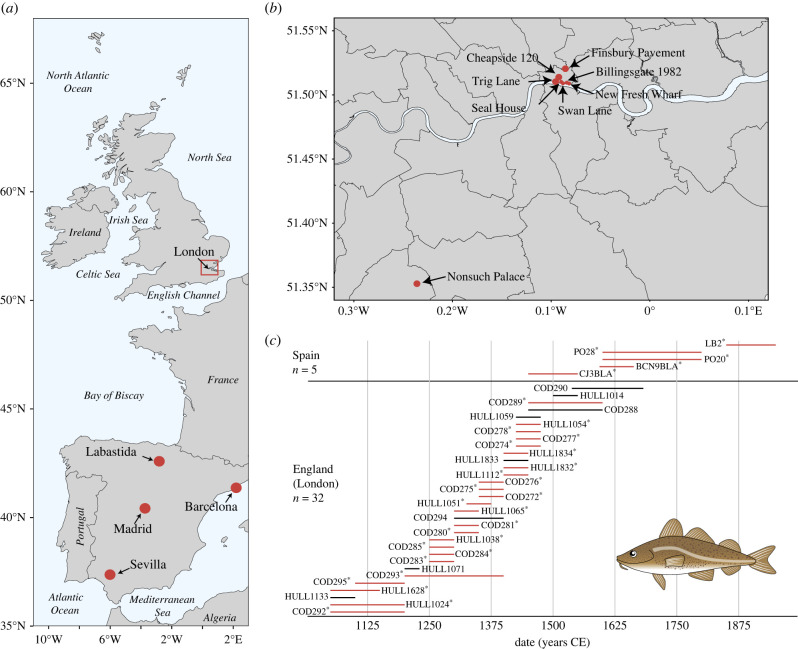
(*a*) Distribution of archaeological Atlantic cod specimens in England (London) and Spain. Spanish locations are highlighted (in red) on the map. (*b*) Detailed distribution (in red) of English archaeological locations in London from which Atlantic cod bones were obtained. (*c*) Date range of the 37 archaeological specimens as estimated based on archaeological context. Samples in red and with ‘asterisk’ (*) yielded sufficient data to allow more detailed genomic assignments. Other samples could only be assigned to major geographical regions (see Results section for explanation). For details regarding the sample codes, see electronic supplementary material, table S1. Fish illustration was drawn by Lourdes Martínez-García. (Online version in colour.)

English samples (*n* = 32) were obtained from eight archaeological locations in London (figure 1*b*; electronic supplementary material, table S1). Based on archaeological evidence and stable isotope analysis, three of the locations (Finsbury Pavement, Seal House and Trig Lane) have previously been inferred to have imported preserved cod [16]. Specimens from the additional five locations (Billingsgate 1982, Cheapside 120, New Fresh Wharf, Nonsuch Palace and Swan Lane) were included to provide a continuous fishing time series from the eleventh to sixteenth–seventeenth centuries CE (figure 1*c*). Seven of the archaeological sites in London were urban when occupied. The eighth location (Nonsuch Palace) was a royal residence originally outside London, which was later surrounded by the modern metropolis. Spanish samples (*n* = 5) were obtained from four different archaeological locations: a monastic-upper class context from La Cartuja (Sevilla, late fifteenth–early sixteenth centuries), an urban context from Plaza de Oriente (Madrid, seventeenth–eighteenth centuries), a context from the fishermen's quarter from Barraques de pescadors (Barcelona; *ca* seventeenth century) and a rural castle (Álava) that acted as a military centre during the nineteenth century (JA Quirós 2022, personal communication; figure 1*a*; electronic supplementary material, table S1). Atlantic cod bones are very rare in Iberian archaeological sites [12]; therefore, the present five Spanish specimens represent those available for this study.

Cranial (articular, premaxilla, frontal, dentary and parasphenoid) and postcranial (vertebra and cleithrum) bones were included (electronic supplementary material, table S1 and figure S2). Cranial bones are more likely to represent local fishing because many preserved fish products were decapitated [32], although, complete fish (and/or preserved fish heads) were sometimes traded over long distances [6,33,34]. Cleithra (which support the pectoral fin just behind the cranium) can be found together with cranial remains, or (if fish were decapitated anterior to this element) with postcranial bones [15]. Here, we considered cleithra belonging among the postcranial bones.

After field collection, all samples were stored dry and unfrozen. Dating of the samples was based on archaeological context. Qualitative date ranges were converted into calendar years as per Orton *et al*. [15] considering an ‘early’ century the first half of that century (e.g. ‘00 to ‘50), ‘mid’ century as ‘25 to ‘75 and ‘late’ century as the second half of that century (e.g. ‘50 to ‘00). The archaeological Atlantic cod samples were morphologically and genetically identified to species.

### (b) aDNA extraction and library preparation

We processed 18 English (London) fish-bone samples in the aDNA laboratory at the University of Oslo [35,36] (electronic supplementary material, table S1). Treatment of samples prior to DNA extraction was according to Ferrari *et al*. [37] and Martínez-García *et al*. [38]. In short, fish bones were UV treated for 10 min per side and milled using a stainless-steel mortar [39]. Milled fish-bone powder was divided in two aliquots per specimen (150–200 mg per aliquot) as starting material for DNA extraction. Genomic DNA was extracted from the fish-bone samples using the mild Bleach treatment and Double-Digestion step (BleDD) protocol [40]. In addition, we added to our initial London assemblage aDNA from 14 English fish-bone samples previously processed at the University of Hull following the protocols in Hutchinson *et al*. [19] (electronic supplementary material, table S1). Three out of the 14 samples were previously inferred to have a southern and central North Sea biological origin (electronic supplementary material, table S1) [19]. Furthermore, we analysed aDNA from five Spanish bone samples previously analysed and processed using a modified protocol of Yang *et al*. [41] at BioArch, University of York. In short, samples were decontaminated with 6% sodium hypochlorite (bleach) for 5 min and then rinsed three times in distilled water. Samples were further UV treated for 10 to 20 min per side. Samples were powdered prior to the addition of a lysis buffer (EDTA) and Proteinase K. Samples were incubated overnight at 50°C while kept in rotation. After incubation, samples were centrifuged to separate bone powder from buffer solution. The supernatant was transferred to an Amicon Ultra-4, Centrifugal Filter Device, 10 000 NMWL tube to concentrate the solution and the Qiagen QiaQuick MinElute™ kit was used for DNA purification. Contamination controls were taken during every step of the extraction and amplification procedure.

Double-indexed blunt-end sequencing libraries were built from 16 or 20 µl of DNA extract from all samples using the double-stranded Meyer-Kircher protocol [42,43] with the modifications listed in Schroeder *et al*. [44] or the single-stranded Santa Cruz Reaction (SCR) protocol using tier four adapter dilutions [45] (see electronic supplementary material, table S1 for specifications). Multiple extraction and negative controls during all library sessions were used to detect possible contamination. All samples were assessed for library quality and concentration using a high-sensitivity DNA Assay on the Bioanalyzer 2100 (Agilent) or with a high-sensitivity NGS Fragment Analysis Kit on the Fragment AnalyzerTM (Advanced Analytical). Successful libraries were sequenced using the Illumina HiSeq 4000 with 150 bp paired reads, or on a Novaseq 6000 with 150 bp paired reads at the Norwegian Sequencing Centre. Sequencing reads were demultiplexed allowing zero mismatches in the index tag and they were processed using PALEOMIX v1.2.13 [46]. Trimming of residual adapter contamination, filtering and collapsing of reads was done using AdapterRemoval v.2.1.7 [47]. Mapping of remaining reads was performed against the gadMor2 reference genome [48,49] using BWA v.0.7.12 [50] with the *backtrack* algorithm, disabled seeding and minimum quality score of 25. aDNA deamination patterns were determined using MapDamage v.2.0.9 [51] and BAM files were indexed with samtools v.1.9 [52].

### (c) Genomic and statistical analysis

**Figure 2.**
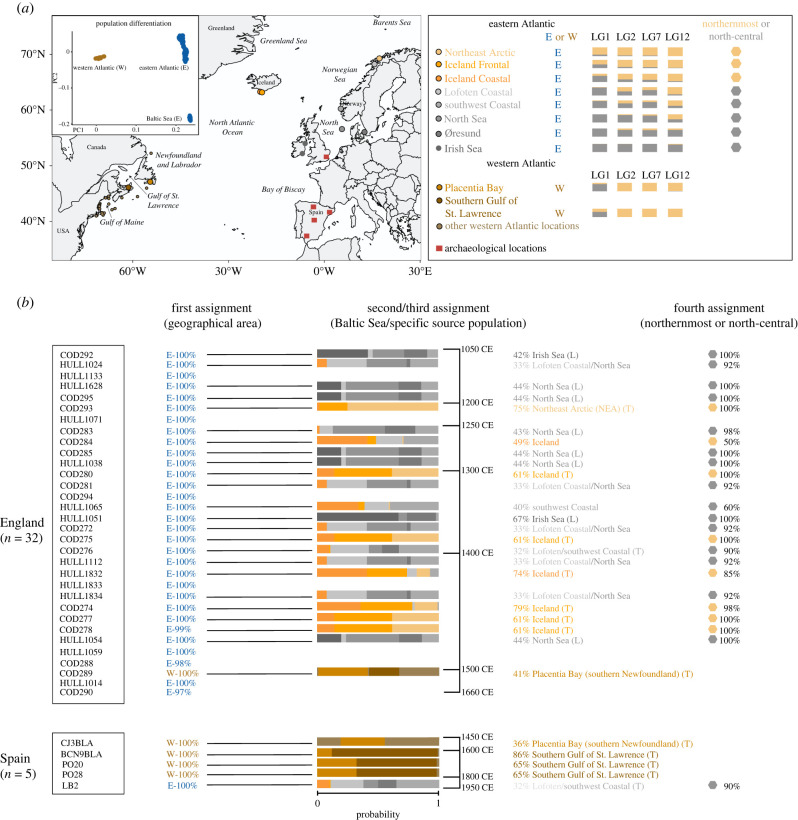
(*a*) Geographical distribution of inversion frequencies of chromosomal inversions in Atlantic cod (LG1, LG2, LG7 and LG12) across the North Atlantic Ocean. The assignment of specific haplotypes to a geographical area is either eastern (E, in blue) or western (W, in brown) Atlantic. The population PCA plot was modified from Ferrari *et al*. [21] and shows the differentiation between the eastern and western Atlantic Oceans, and the Baltic Sea (in blue as it is located within the eastern Atlantic Ocean). Specific alleles associated with a northernmost composite genotype distribution are assigned in orange. Alleles associated with a more temperate north-central genotype distribution are assigned in grey. The archaeological locations are indicated with red squares. The modern populations used as possible source populations of our ancient specimens are located in the map. (*b*) First genomic assignment: overall percentages (%) represent the minimum probability obtained to be from either the eastern or the western Atlantic Ocean. Second/third genomic assignment: source population percentages (%) represent the highest probability to be assigned to a specific modern location (electronic supplementary material, tables S2–S4). Iceland assignment is obtained by adding the probabilities of both frontal and coastal Icelandic ecotypes. Only two locations from the western Atlantic region have divergent inversion genotype frequencies; thus, they have a specific assignment colour (Placentia Bay and Southern Gulf of St. Lawrence). Other western Atlantic locations are represented in light brown colour (electronic supplementary material, tables S3 and S4). The approximate age (CE) of the specimens is indicated on the right side of the bar plot. Specific time periods are found in figure 1, the electronic supplementary material, table S1 and figure S2. A local (L) or traded (T) assignment follows putative source population. Specimens are considered to be of local origin with considerable North Sea or Irish Sea assignments, specimens are considered to have been obtained through trade with significant Northeast Arctic, Norwegian coast, Iceland or western Atlantic assignments. Individuals with ambiguous origin (i.e. HULL1024, COD281, COD272, HULL1112 and HULL1834) or with a northernmost or north-central origin below 75% probability (i.e. COD284 and HULL1065) are not identified as local or traded in this chronology. Individuals COD276 and LB2 are identified as traded specimens as their likely origin is a remote population: Norwegian coast (Lofoten or southwest). Fourth assignment: percentages (%) representing either the northernmost or north-central genotype distribution after adding the scaled probabilities of selected source populations. For details of the sample codes, see electronic supplementary material, table S1. (Online version in colour.)

To infer the biological origin (source population) of the archaeological samples of Atlantic cod, we followed a hierarchical procedure (figure 2). First, we used BAMscorer, a software that can assign low-coverage sequences to biological populations following the methodology described in Ferrari *et al*. [21]. This approach creates databases of spatially divergent genetic data, analysing high-coverage whole-genome data of modern individuals of known provenance. These modern specimens were obtained from Barth *et al*. [53] and Pinsky *et al*. [24] and comprise 276 Atlantic cod individuals that represent three broad geographical regions of the species' range: the western Atlantic Ocean, the eastern Atlantic Ocean and the Baltic Sea. All of these regions are genetically differentiated and represent potential sources of distant-water fishing and fish trade over the chronology of our study [24,53]. Three major genetic clusters (here named western Atlantic, eastern Atlantic and Baltic Sea) can be identified and genetically assigned using genome-wide data (excluding the four large chromosomal inversions in Atlantic cod: LG1, LG2, LG7 and LG12, figure 2*a*). Based on such genome-wide differentiation, the first assignment was used to determine an overall eastern or western Atlantic Ocean origin. Subsequently, using a similar approach, all specimens first assigned to a putative eastern Atlantic region were analysed to determine a possible Baltic Sea origin [21].

Then, again using the BAMscorer pipeline [21], we identified specific individual genotypes of the four large chromosomal inversions in our low-coverage ancient samples. These inversions have highly divergent genetic haplotypes [22,27–30] whose spatial distributions show elevated differentiation between different Atlantic cod ecotypes and spawning regions within the western and eastern Atlantic cluster [22–25]. Therefore, these genotypes can be used to further assign each ancient specimen to a more specific source population within these three genome-wide clusters (electronic supplementary material, table S2). For this, we used the binomial sampling method as per Star *et al*. [6], to infer the overall probability of obtaining a specific composite inversion genotype based on the inversion frequencies of the specific source populations [22–25]. In short, this approach investigates if ancient individuals statistically differ in their affinity towards a particular modern population based on their inversion genotypes. The probability of obtaining an individual inversion genotype follows a binomial distribution given the underlying allele frequencies of a modern population. Since the inversions are located on different chromosomes, we can assume independence between inversion loci. We then calculated the overall probability of obtaining a composite ancient inversion genotype—based on the modern populations’ respective allele frequencies—as a measure of an individual's affinity toward a specific population (electronic supplementary material, table S2).

Given the low-coverage data, we used the negative controls from the library sessions as a baseline to include or exclude specimens for specific genomic assignments. We included those samples with greater than 2000 aligned reads and at least one magnitude more of nuclear coverage and endogenous DNA than our negative controls. Subsequently, we only infer the genotype of samples with at least three chromosomal inversions with greater than 75% probability (see details in electronic supplementary material, tables S1 and S2). Comparative modern inversion frequency data were compiled for a range of different populations [22–25]. From the eastern Atlantic, we included: the Northeast Arctic, the Norwegian Coast (Lofoten and the southwest), the North Sea, the Irish Sea, Øresund and Iceland (both coastal and frontal ecotypes, which differ in their migratory behaviour). Western Atlantic populations included a number of populations south and north of Newfoundland (figure 2*a*; electronic supplementary material, tables S3 and S4). The highest specific assignment probabilities are reported as percentages (%) in tables and figures, representing the confidence with which one individual is assigned to a specific population (i.e. 75%). In the event of similar assignment probabilities for more than one population (e.g. 50% and 50%), both populations are reported as the putative origin of the individual (i.e. Norwegian Coast or North Sea, see electronic supplementary material, tables S3 and S4 for specific details of assignment percentages). Finally, we recognized that most eastern Atlantic individuals could be further classified with high confidence towards two spatially distinct groups; an overall assignment to a northernmost (Northeast Arctic and Iceland) or north-central (Norwegian Coast, North Sea, Irish Sea and Øresund) distribution (by adding the scaled probabilities of source populations; see details in the electronic supplementary material, tables S3 and S4).

We performed a Fisher's exact test to assess for the existence of an association between bone element and specimen origin (i.e. sourced through trade versus local landings from the North Sea or Irish Sea). The test was implemented in the *stats* and *ggstatsplot* libraries in R [54,55] using 22 samples that were assigned to a specific source population (figure 2*b*; electronic supplementary material, S3). We excluded samples with a northernmost or north-central assignment with less than 75% probability and samples with an indistinguishable origin (see Results section for more details).

## 3. Results

We sequenced 37 specimens and obtained a total of approximately 342 million paired reads, approximately 9 million aligned reads and endogenous DNA content between less than 0.01% and 34% (electronic supplementary material, table S2). Sequencing reads showed the patterns of DNA fragmentation and deamination rates that are consistent with those of authentic aDNA (electronic supplementary material, figure S1). We successfully assigned these 37 sequenced specimens to one of the two broad geographical areas (eastern or western Atlantic Ocean), finding a total of five samples (England = 1, Spain = 4) from the western Atlantic and 32 specimens (England = 31, Spain = 1) from the eastern Atlantic (figure 2*b*; electronic supplementary material, tables S1 and S2). Within the eastern Atlantic specimens, we found two that could be assigned to the Baltic Sea at 54% and 98% probability (electronic supplementary material, table S2), however, because of low numbers of obtained sequence reads such assignments were not considered for our final chronology. Subsequently, we assigned a total of 29 out of the 37 samples (England = 24, Spain = 5) to a more specific source population based on the assignment probabilities of their composite inversion genotypes (electronic supplementary material, table S2; figure 2*b*). For samples assigned to a specific source population, we could identify 102 out of 116 inversion genotypes with more than 95% probability (electronic supplementary material, table S2). Specific assignments are dependent on the source populations provided for comparison, which can result in low probability assignments as several populations can share similar inversion genotype frequencies (e.g. less than 50% probability; figure 2*b*). In agreement with postcranial bones being commonly assigned to non-local sources, we found a statistically significant association between the bone element (cranial or postcranial) and the origin (local or traded, respectively) of the specimen (*p* = 0.01; electronic supplementary material, table S1 and figures S2 and S3).

For London, of the 31 specimens assigned to the eastern Atlantic Ocean, 24 passed the initial quality baselines required for specific assignment analysis and had sufficient data for estimating genotypes of inversion loci. We assigned 15 specimens to a north-central haplotype group (with greater than 60% probability). This includes eight specimens with a possible source population like the North Sea or the Irish Sea (42–67% probability), one specimen with a southwest Norwegian Coast origin (40% probability) and six specimens with indistinguishable associations to the Norwegian Coast (both Lofoten and southwest) and the North Sea (32–33% probability). Nonetheless, these within-group assignments are not strongly supported as the inversion frequency differences of the reference populations are limited (figure 2*a*). Similarly, we assigned seven specimens to a northernmost composite genotype group (with greater than 85% probability), where we found one specimen possibly coming from northern Norway (Northeast Arctic, 75% probability) and six specimens likely coming from Iceland (61–79% probability). We calculated an overall Icelandic origin by adding the probabilities of being Icelandic frontal or coastal ecotypes. The genomic distinction between Icelandic cod and Northeast Arctic cod is predominantly driven by the higher frequency of north-central genotypes (in grey figure 2*a*) for the chromosomal inversion LG01 in Iceland [22]. However, similar inversion frequencies (for LG01) between deep water Icelandic cod and Northeast Arctic cod have been reported [56]. Considering such similarities, the assignments to Iceland or the Northeast Arctic should be taken with caution. Moreover, we found an unreliable assignment for one specimen to a northernmost or north-central composite genotype group (with 50% probability), resulting in an Icelandic assignment with low confidence (49%; figure 2*b*; electronic supplementary material, table S3). Our specific genomic assignments further agree with a presumed local origin of two specimens included in our final chronology previously used as ‘local’ (control) samples for aDNA analysis in Hutchinson *et al*. [19] (electronic supplementary material, table S1). These samples (HULL1038 and HULL1065) have a north-central haplotype group distribution with 60% and 100% probability, respectively. In combination with the significant association between the bone element (cranial or postcranial) and the origin (local or traded) of a specimen, these bones are therefore likely of a local origin. Finally, as noted above, we assigned one London specimen (dated between the late fifteenth and sixteenth centuries) to a western Atlantic origin (with 100% probability) including a possible low confidence assignment to Placentia Bay (41% probability; figure 2*b*; electronic supplementary material, table S3).

For Spain, we found four specimens assigned to the western Atlantic and one specimen assigned to the eastern Atlantic (figure 2*b*). The assignments to the western Atlantic (with 100% probability) tentatively included source populations along southwestern Newfoundland (Placentia Bay) or the Gulf of St. Lawrence (with 36–86% probability). We assigned the eastern Atlantic specimen (with 90% probability) to a north-central composite genotype group which includes the Norwegian Coast as a putative origin (indistinguishable association to the Lofoten and southwest coast with 32–33% probability; figure 2*b*; electronic supplementary material, table S4).

## 4. Discussion

We have used a novel genomic assignment approach to identify the biological source of individual archaeological Atlantic cod specimens from England and Spain. With high confidence, we assigned fish remains to a large-scale geographical origin (up to 100% assignment probability) and genotype groups within regions (greater than 85% assignment probability). With moderate to low confidence (less than 86% assignment probability), we tentatively identified several of the specimens to more specific spatially constrained populations. Below we describe the resulting spatio-temporal patterns observed in England and Spain, and consider the impact these findings have on our understanding of the globalization of marine fisheries over the last millennium.

### (a) London: an increasingly North Atlantic trade

Previous zooarchaeological evidence and stable isotope data [15,16] implied that Atlantic cod trade stemmed predominantly from local fisheries during the eleventh to twelfth centuries, after which longer distance imports appeared during the thirteenth to fourteenth centuries. Our individual assignments provide confident aDNA evidence that supports this chronology, with fish assigned to northern Atlantic regions appearing in increasing frequency over time. Assignments to specific source populations are often associated with lower individual probabilities (approx. 32% probability); therefore, these should be considered as indicative only. Interestingly, our assignment analysis indicates that imported Atlantic cod dated between the thirteenth and the fourteenth centuries derived not only from northern Norway but possibly from Iceland (figure 2*b*). According to historical evidence, Iceland first became a major supplier of dried cod to England during the fifteenth century, when fishermen and merchants from England and Germany first defied the Norwegian royal monopoly on trade with Iceland [57,58]. However, exports of *stockfish* from Iceland via Norway commenced *ca* 1300 CE or earlier [59,60]. Our results are consistent with this chronology. Icelandic cod in medieval London would probably have reached England via Bergen, on Norwegian, English, and/or Hanseatic ships that are known to have traded between Norway and ports of the English east coast [61,62].

Furthermore, England's participation in the western Atlantic cod fisheries expanded in south-eastern Newfoundland during the late sixteenth century (figure 2*b*; electronic supplementary material, table S4) [63]. Therefore, the observation of a specimen from the western Atlantic dated to the late fifteenth to the sixteenth centuries currently represents one of the earliest known genetic examples of this trans-Atlantic expansion. This chronology is consistent with existing knowledge regarding the emergence of trans-Atlantic cod trade, although, as discussed further below, English catches in North America were often destined for southern Europe rather than home markets like London [14,64].

### (b) Spain: an increasingly trans-Atlantic trade

Similar to the single late fifteenth- to sixteenth-century London cod bone, our findings in Spain are consistent with known western Atlantic fishing expansion of the early modern period [65]. In a historical context, the Basques and Galicians provided Atlantic cod for Spain throughout the sixteenth century [64–66]. The fifteenth- to sixteenth-century sample from Spain assigned to waters of the western Atlantic is consistent with historical sources, which indicate that Basque fishermen (from Spain and France) and Galicians acquired fish from southern to western Newfoundland (i.e. Placentia Bay, the Gulf of St. Lawrence, St. Pierre and Miquelon) to fulfil the demand for Atlantic cod in Spain [65,67–70]. The three later specimens (seventeenth century and seventeenth to eighteenth centuries) could have derived from Spanish fishermen operating in Newfoundland [66,70] or as trade items with English or French fishermen that had been operating in these grounds since the sixteenth century. In fact, the English engaged in unofficial trade even when political hostilities disrupted relations with Spain [64]. These specimens may thus relate to the triangular trade involving an exchange of Atlantic cod from the western North Atlantic for wine and salt from southern Europe [14]. By the late nineteenth to early twentieth century, dried Atlantic cod from across the Norwegian waters could have been used to provision the military centre in Álava (JA Quirós 2022, personal communication). Our most recent Spanish sample originating from the Norwegian Coast (figure 2*b*; electronic supplementary material, table S4) is therefore consistent with the supply of northern European air-dried Atlantic cod since the eighteenth century [71,72].

## 5. Conclusion

Altogether, our results provide genetic evidence for an expanding trade and increasing demand for marine fish leading to the exploitation of a great diversity of distant-water sources already in the Middle Ages. Our evidence also tracks the culmination of the marine fisheries extension with European exploitation of the western Atlantic fishing grounds around Newfoundland, starting in the sixteenth century. Our findings emphasize the utility of whole-genome sequencing and aDNA methods to describe the increasing demand for Atlantic cod for European societies during medieval and post-medieval periods. We expect that the inclusion of more archaeological sites and larger sample sizes through space and time will reveal additional patterns about those populations targeted for long-distance trade in the past and possibly provide greater insight about which populations have experienced long-term impacts of fisheries. Overall, our results corroborate and significantly increase existing knowledge about the globalization of marine fisheries and fish trade in medieval and early modern times.

## Data Availability

The raw reads for the ancient specimens are released under the ENA accession number PRJEB52865. This manuscript is available as a pre-print in BioRxiv at https://www.biorxiv.org/content/10.1101/2022.06.03.494519v1 [73]. The data are provided in the electronic supplementary material [74].
